# Biomechanical Consequences of Nail Insertion Point and Anterior Cortical Perforation for Antegrade Femoral Nailing

**DOI:** 10.1155/2020/5878607

**Published:** 2020-12-18

**Authors:** Michael Ching, Aaron Gee, Christopher Del Balso, Abdel Lawendy, Emil H. Schemitsch, Radovan Zdero, David Sanders

**Affiliations:** ^1^Department of Surgery (Division of Orthopaedic Surgery), Western University, London, ON, Canada; ^2^Orthopaedic Biomechanics Lab, Victoria Hospital, London, ON, Canada; ^3^Department of Mechanical and Materials Engineering, Western University, London, ON, Canada

## Abstract

This biomechanical study assessed the influence of changing antegrade cephalomedullary nail insertion point from anterior to neutral to posterior locations relative to the tip of the greater trochanter with or without anterior cortical perforation in the distal femur. Artificial osteoporotic femurs and cephalomedullary nails were used to create 5 test groups each with 8 specimens: intact femur without a nail or perforation, anterior nail insertion point without perforation, neutral nail insertion point without perforation, posterior nail insertion point without perforation, and posterior nail insertion point with perforation. Nondestructive biomechanical tests were done at 250 N in axial, coronal 3-point bending, sagittal 3-point bending, and torsional loading in order to measure overall stiffness and bone stress. The intact femur group vs. all femur/nail groups had lower stiffness in all loading modes (*p* ≤ 0.018), as well as higher bone stress in the proximal femur (*p* ≤ 0.027) but not in the distal femur above the perforation (*p* = 0.096). Compared to each other, femur/nail groups only showed differences in sagittal 3-point bending stiffness for anterior and neutral vs. posterior nail insertion points without (*p* ≤ 0.025) and with perforation (*p* ≤ 0.047). Although it did not achieve statistical significance (*p* ≥ 0.096), moving the nail insertion point from anterior to neutral to posterior to posterior with perforation did gradually increase bone stress by 45% (proximal femur) and 46% (distal femur). No femur or hardware failures occurred. Moving the nail insertion point and the presence of a perforation had little effect on stiffness, but the increased bone stress may be important as a predictor of fracture. Based on current bone stress results, surgeons should use anterior or neutral nail insertion points to reduce the risk of anterior cortical perforation.

## 1. Introduction

Hip fractures are a common problem in the elderly because of the increased prevalence of osteoporosis in this population. In women, the proportion of hip fractures that also involve the trochanteric region rises with age from 35% (55-59 years old) to 51% (84 years old and above) [1]. Conversely, in men, the proportion of hip fractures that also involve the trochanteric region slightly decreases with age from 47% (55-59 years old) to 44% (84 years old and above) [1]. However, women are 2.5 times as likely as men to experience a hip fracture [2]. In any case, 90% of hip fractures occur in patients over 70 years of age, most of which occur due to a fall from a standing height; the force from such a fall exceeds the femoral strength of an older individual by about 50% [3]. The mortality rate of fractures associated with osteoporosis ranges from 15 to 30%, while 50% of osteoporotic women with hip fractures develop disability, which may lead to institutionalization [4].

Clinically, cephalomedullary nails are used commonly to treat hip fractures, although other implants (e.g., arthroplasties, cannulated screws, and sliding hip screws) may be used depending on the specific hip fracture pattern or the surgeon's preference. The technique involves inserting an antegrade nail into the femoral medullary canal which is secured distally using locking screws, as well as insertion of a lag screw into the femoral neck and head. The small incision required for nail insertion can reduce blood loss, shorten operative times, lower malunion rates, and reduce fracture collapse compared to sliding hip screws [5–8]. Moreover, clinical outcomes for cephalomedullary nailing of hip fractures have been good with an 85% rate of maintaining fracture stability until union or death [9], a less than 3% rate of failure of the implant itself [10], and only a 1.4% rate of lag screw cutout and a 4% rate of fracture collapse into varus > 10° with an immediate full weight-bearing rehab plan [11].

Biomechanically, cephalomedullary nails have been assessed in a number of in vitro studies using cadaveric or artificial femurs in order to determine the optimal lag screw position in the femoral head [12], the effect of using a static lag screw vs. a dynamic lag screw that moves within the nail to compress the fracture [13], the force required to insert the nail into the trochanter for devices with varying amounts of curvature [14], and the relative performance of nails vs. sliding hip screws [15]. Moreover, there is one biomechanical report on insertion of a long antegrade noncephalomedullary nail (i.e., no lag screw) at the piriformis fossa vs. the tip of the greater trochanter vs. lateral to the tip of the greater trochanter [16], while another biomechanical investigation examined insertion of a short antegrade cephalomedullary nail at the piriformis fossa vs. the tip of the greater trochanter [17]. However, no prior biomechanical studies have considered the effect of moving the cephalomedullary nail insertion point within the sagittal plane, which may be an important variable in accidentally causing anterior cortical perforation in the distal femur [18, 19]. Although the overall rate of perforation is less than 1% [20, 21], this will need to be addressed either by restricting the patient to partial weight bearing [22, 23] or applying lateral locking plates [23] because of the impending risk of displaced supracondylar fracture requiring revision surgery [24].

Therefore, this will be the first biomechanical study to analyze the effect of moving the antegrade cephalomedullary nail insertion point from anterior to neutral to posterior locations relative to the tip of the greater trochanter with or without an anterior cortical perforation in the distal femur. The hypothesis is that nail insertion point and the presence of perforation will alter the initial postoperative biomechanical stability of the nailing technique.

## 2. Methods

### 2.1. General Approach

This study assessed the biomechanical effect of nail insertion point and anterior cortical perforation in the distal femur for antegrade femoral nailing in a postunion, rather than a postoperative, scenario. To do so, 8 intact artificial femurs were first tested biomechanically for stiffness and stress to provide baseline values, distributed into 4 groups of 2 femurs each based on the rank order method (i.e., the femur with the highest value was paired with the femur with the lowest value, the femur with the 2nd highest value was paired with the femur with the 2nd lowest value, etc.), and then randomly assigned to one of 4 implant groups. Thus, there were 5 test groups of 8 specimens each, namely, 1 intact femur group plus 4 femur/nail groups with varying nail insertion points with or without cortical perforation. All biomechanical test fixtures, loading regimes, measurement techniques, data analyses, and statistical analyses were based on previous protocols [12, 13, 15, 25–29].

### 2.2. Surgical Procedures

Thirty-two left-sided medium-sized artificial femurs designed to mimic osteoporosis were obtained (Model 3503-118; Sawbones, Vashon, WA, USA) [30], each having a premachined hollow medullary canal diameter of 18 mm, simulated cortical density of 1.3 g/cm^3^, and simulated cancellous density of 0.16 g/cm^3^; these analogs were previously biomechanically validated against osteoporotic cadaveric femurs with good agreement for axial, 4-point bending, and torsional stiffness, as well as screw pullout force [31].

Three Gamma3 R1.5 titanium nails (Stryker Canada, Hamilton, ON, Canada) (distal diameter, 10 mm; total length, 400 mm) and titanium lag screws (diameter, 10.5 mm; total length, 100 mm) were obtained that would create a neck-shaft angle of 125°. A set screw was tightened to the lag screw to lock the proximal construct. At the distal femur, the more proximal locking screw (diameter, 5 mm; length, 55 mm) and the more distal locking screw (diameter, 5 mm; length, 75 mm) were both fully threaded. Implants were able to be used multiple times by random assignment to artificial femurs, since all later biomechanical tests were at low nondestructive loads.

**Figure 1:**
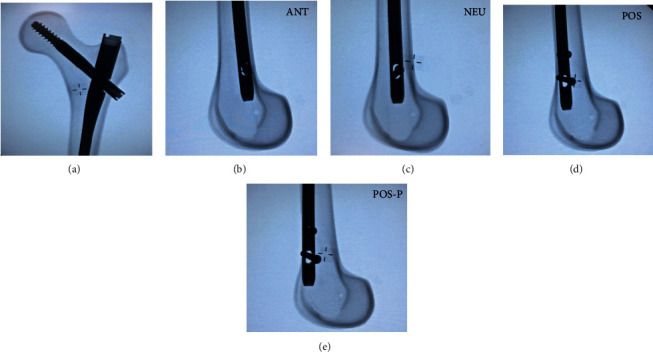
Radiographic views of test groups: (a) typical coronal view of the proximal femur, nail, and lag screw for all implanted groups; (b) anterior nail insertion without perforation; (c) neutral nail insertion without perforation; (d) posterior nail insertion without perforation; (e) posterior nail insertion with perforation. An intact (INT) group, not shown, was also used as the control.

Five test groups were then created, each with 8 femurs. INT was the intact femur group without implants or perforations. ANT had a nail insertion point anterior to the midline of the proximal canal and no perforation (Figures 1(a) and 1(b)). NEU had a nail insertion point neutral (i.e., in line) to the midline of the proximal canal and no perforation (Figures 1(a) and 1(c)). POS had a nail insertion point posterior to the midline of the proximal canal and no perforation (Figures 1(a) and 1(d)). POS-P had a nail insertion point posterior to the midline of the proximal canal, but with an anterior cortical perforation in the distal femur (Figures 1(a) and 1(e)). Nailing was done according to the manufacturer after identifying the insertion point. On anteroposterior radiographic views, the nail insertion point was seen at the greater trochanter, while on lateral radiographic views, the nail insertion point was seen with respect to the midline of the proximal canal. The entry reamer was advanced, followed by the nail. A lag screw was then inserted aiming for the center of the femoral head and advanced appropriately. A set screw was then placed in locking configuration. Two distal screws were then inserted freehand under fluoroscopic guidance using the perfect circle freehand technique.

**Figure 2:**
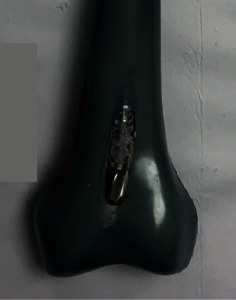
Eccentric reaming of the anterior cortex using a 10 mm diameter reamer created the anterior cortical perforation of the distal femur for the POS-P nail group. The reamer was advanced further than shown, until the full size of the perforation was made.

Anterior cortical perforations in the POS-P group were created in the distal femur by eccentric reaming of the anterior cortex of the distal femur, similar to that caused by the reamer-irrigator-system (Figure 2) [32]. A guidewire was advanced retrograde through the anterior cortex of the distal femur into the shaft. A 10 mm diameter reamer was then passed over the guidewire and advanced until the cortex was fully perforated. Perforations were along the shaft midline, ended just above the intercondylar notch, and were 10 mm wide and 60 mm long.

### 2.3. Axial Tests

**Figure 3:**
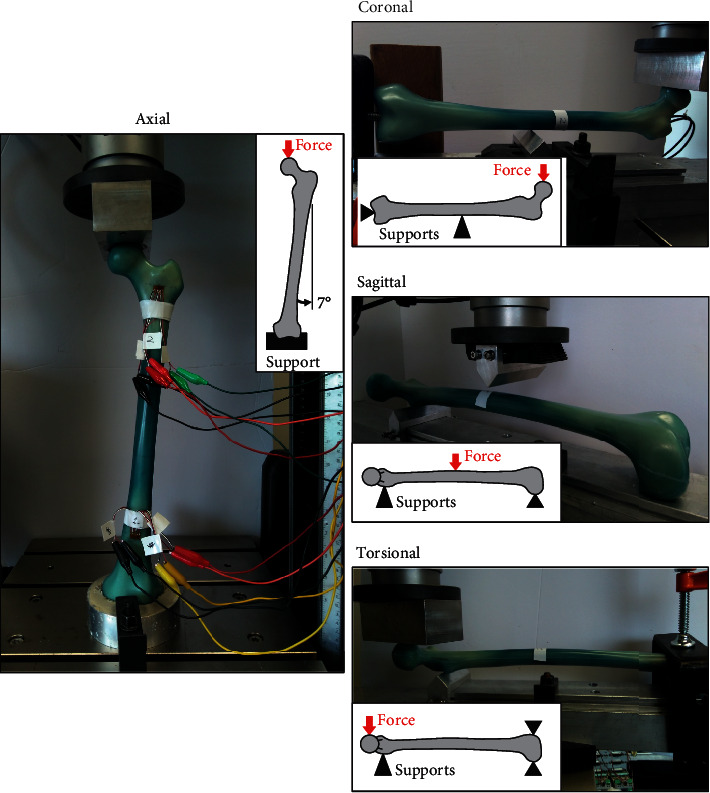
Biomechanical loading modes for axial, coronal, sagittal, and torsional tests. Only an intact (INT) specimen is shown, but the setups were the same for all test groups. Rosettes and associated wiring were only used during axial tests.

All axial tests (as well as those described below) were done at room temperature using a mechanical tester (Instron 5967, Norwood, MA, USA) equipped with its own load cell (±30 kN range and ±0.5% accuracy) and displacement transducer (1140 mm range and ±0.05% accuracy). Each intact and implanted femur was aligned in 7° of adduction in the coronal plane and aligned vertically in the sagittal plane to replicate the one-legged stance phase of walking (Figure 3). Distally, the condyles rested on top of a rigidly clamped and tailor-made cement block (Flowstone, King Packaged Materials Company, Burlington, ON, Canada) that matched the condylar geometry perfectly, thereby simulating the tibial plateau. Proximally, the femoral head was inserted into a smooth metal cup mimicking the acetabulum. A vertical force was then applied to the superior surface of the femoral head through the metal cup using force control (preload, 25 N; max load, 250 N; load sustain, 120 s; load rate, 10 N/s). The slope of the initial rise of the force-displacement graph (i.e., 25 to 250 N) was defined as axial stiffness, while the coefficient of determination was *R*^2^ > 0.96 indicating the high linearity of the graph and that no gross damage was done to the femur or implant.

**Figure 4:**
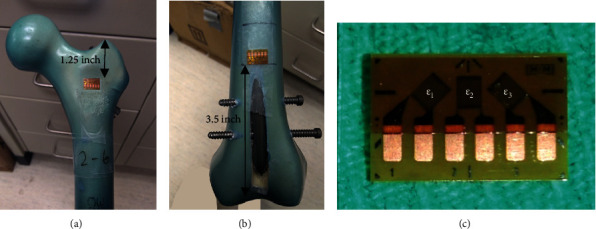
Rosette locations: (a) proximal rosette, (b) distal rosette, and (c) close-up of rosette with linear strain gages *Ɛ*_1_, *Ɛ*_2_, and *Ɛ*_3_. Wire leads are not shown so rosettes are clearly visible. Only a typical POS-P specimen (i.e., with perforation) is shown, but even for INT, ANT, NEU, and POS groups (i.e., without perforation), the rosettes were at the same corresponding locations to make direct comparisons of bone stress possible.

Rosette strain gage readings were also collected during axial tests, since this is a long established technique of nondestructively assessing local bone stresses leading to potential bone failure; however, rosette readings were only recorded for axial tests, since this is the loading mode most often assessed for potential bone failure by biomechanical studies on femur fixation. Each intact and implanted femur was equipped with 2 rosettes (Model CEA-06-062UR-350, Vishay Micro-Measurements, Raleigh, NC, USA), which were each composed of 3 linear strain gages arranged in a “rectangular” 0°-45°-90° pattern. The proximal rosette was located on the anterior surface midway between the greater and lesser trochanters (i.e., the distance from the rosette's top edge to the greater trochanter was 1.25 inches) (Figure 4(a)), whereas the distal rosette was located 10 mm above the anterior perforation for perforated femurs or at the exact corresponding location for nonperforated femurs (i.e., the distance from the rosette's bottom edge to the intercondylar notch was 3.5 inches) (Figure 4(b)). Wire leads were soldered to the rosettes, secured to the femur using tape, and connected to an 8-channel data acquisition system via a quarter bridge Wheatstone configuration (Cronos-PL, IMC Mess-Systeme GmbH, Berlin, Germany), which was linked to a computer for data storage and analysis with dedicated software (Famos v5.0, IMC Mess-Systeme GmbH, Berlin, Germany). The manufacturer-provided gage factor of 2.1 was used, which is an index for strain sensitivity at a particular temperature, i.e., ratio of resistance change to strain change. Each rosette reading was actually composed of 3 linear strain readings (Figure 4(c)) that were averaged for the middle 90 s of the 120 s load sustain period and then converted to a final Von Mises stress for each rosette; this represents the stress magnitude but not its type (i.e., tensile or compressive) or 3D direction (i.e., *x*, *y*, and *z* directional components of the magnitude). To do so, the experimental values of *Ɛ*_1,2,3_ = measured linear strain readings, *E* = artificial cortical bone elastic modulus for this particular femur = 6 GPa [30], and *ν* = artificial cortical bone Poisson′s ratio for this particular femur = 0.26 [30] were used to compute Von Mises stress for each “rectangular” rosette with these formulas:

(1) $$\begin{array}{c} S_{\text{VM}}=\text{Von Mises stress}=\sqrt{S_{\text{MAX}}^{2}+S_{\text{MIN}}^{2}-S_{\text{MAX}}S_{\text{MIN}}},\\ S_{\text{MAX}}=\text{maximum principal stress}=\frac{\text{E}}{2}\left[\frac{\left({Ɛ}_{1}+{Ɛ}_{3}\right)}{\left(1-\nu \right)}+\frac{\sqrt{2}}{\left(1+\nu \right)}\sqrt{{\left({Ɛ}_{1}-{Ɛ}_{2}\right)}^{2}+{\left({Ɛ}_{2}-{Ɛ}_{3}\right)}^{2}}\right],\\ S_{\text{MIN}}=\text{minimum principal stress}=\frac{\text{E}}{2}\left[\frac{\left({Ɛ}_{1}+{Ɛ}_{3}\right)}{\left(1-\nu \right)}-\frac{\sqrt{2}}{\left(1+\nu \right)}\sqrt{{\left({Ɛ}_{1}-{Ɛ}_{2}\right)}^{2}+{\left({Ɛ}_{2}-{Ɛ}_{3}\right)}^{2}}\right]. \end{array}$$

### 2.4. Coronal Tests

Each intact and implanted femur was placed horizontally into a 3-point bending test jig with the femoral head facing upwards to mimic side loading at about midshaft that might occur during an injury event (Figure 3). Specifically, a metal support triangle was placed under the shaft at a distance of 190 mm from the intercondylar notch, a support bolt was inserted superficially into the distal end of the intramedullary canal, and a support block was lightly pressed up against the posterior condylar surface to prevent femur rotation. A vertical force was then applied to the medial surface of the femoral head through a smooth metal cup using force control (preload, 25 N; max load, 250 N; load rate, 10 N/s). The slope of the initial rise of the force-displacement graph (i.e., 25 to 250 N) was defined as coronal stiffness, while the coefficient of determination was *R*^2^ > 0.99 indicating the high linearity of the graph and that no gross damage was done to the femur or implant. No rosette readings were collected.

### 2.5. Sagittal Tests

Each intact and implanted femur was positioned horizontally into a 3-point bending test jig with the femoral head facing sideways to simulate front loading at midshaft that might happen during an injury event (Figure 3). Specifically, a metal support triangle was placed just proximal to the lesser trochanter, while the posterior surface of the condyles rested freely on top of a metal plate, so that the distance between the proximal and distal supports was 400 mm. A vertical force was then applied to the anterior surface of the femoral shaft through a metal triangle located at about midshaft (i.e., 203 mm from the proximal support triangle) using force control (preload, 25 N; max load, 250 N; load rate 10 N/s). The slope of the initial rise of the force-displacement graph (i.e., 25 to 250 N) was defined as sagittal stiffness, while the coefficient of determination was *R*^2^ > 0.99 indicating the high linearity of the graph and that no gross damage was done to the femur or implant. No rosette readings were collected.

### 2.6. Torsional Tests

Each intact and implanted femur was placed horizontally into a test jig with the femoral head facing sideways to mimic femoral shaft rotation during physiological activities (Figure 3). Specifically, a metal support triangle was placed just proximal to the lesser trochanter, the posterior surface of the condyles rested on top of a metal plate, and the anterior surface of the condyles was clamped using a metal plate to prevent condylar rotation, so that the distance between the proximal and distal supports was 400 mm. A vertical force was then applied to the anterior surface of the femoral head through a smooth flat metal block using force control (preload, 25 N; max load, 250 N; load rate, 10 N/s). Note that, in addition to pure rotation around the shaft, this loading setup did produce some minor bending around the metal triangle support. The slope of the initial rise of the force-displacement graph (i.e., 25 to 250 N) was defined as torsional stiffness, while the coefficient of determination was *R*^2^ > 0.99 indicating the high linearity of the graph and that no gross damage was done to the femur or implant. No rosette readings were collected.

### 2.7. Statistical Analysis

For *α* = 0.05 (i.e., 5% chance that the null hypothesis is true), *β* = 0.8 (i.e., 80% statistical power for adequate sample size), and *σ* = 0.1 (i.e., 10% variability between artificial femurs) [33, 34], a minimum sample size of 7 femurs per test group was calculated to be able to detect a 15% difference between the means of the test groups. This was deemed adequate, since previous data showed that pathologic fracture risk increased above a 35% reduction in axial, bending, or torsional stiffness [35]. Thus, to be safe, 8 femurs per test group were used. Statistical analysis to compare stiffness and stress measurements of the 5 test groups was done using one-way ANOVA (analysis of variance) with the SPSS software (SPSS Inc., Chicago, IL, USA) to determine if there was any statistical difference using *α* = 0.05 as the criterion. So, if ANOVA showed *p* > 0.05, this meant there was no statistical difference between any test groups for that measurement, and the ANOVA *p* value was reported. But, if ANOVA showed *p* ≤ 0.05, this meant there was a statistical difference somewhere; then, the Tukey's honesty significant difference method was used to identify exactly which pairwise comparisons were statistically different or nondifferent, and the Tukey *p* values were reported.

## 3. Results

**Figure 5:**
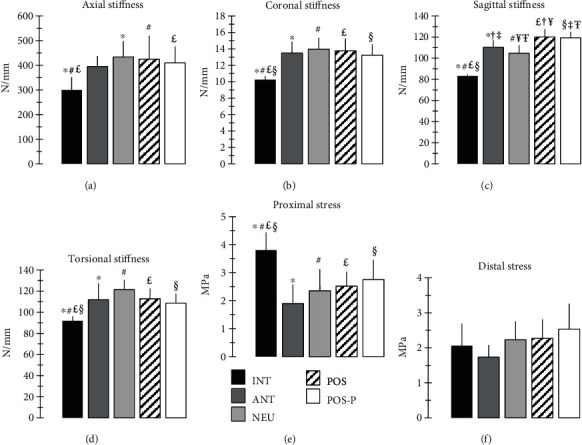
Stiffness and stress results: (a) axial stiffness, (b) coronal 3-point bending stiffness, (c) sagittal 3-point bending stiffness, (d) torsional stiffness, (e) proximal stress, and (f) distal stress. INT: intact femur; ANT: anterior nail insertion without perforation; NEU: neutral nail insertion without perforation; POS: posterior nail insertion without perforation; POS-P: posterior nail insertion with perforation. Each bar represents the mean ± 1 standard deviation. Symbols indicate all statistical differences detected for pairwise comparisons (*p* ≤ 0.05).

Stiffness and stress data are shown (Figure 5). Note also that no femurs, nails, lag screws, or locking screws showed any signs of fracture or failure during any tests.

For axial stiffness, the INT group was less stiff than NEU, POS, and POS-P nail groups (0.002 ≤ *p* ≤ 0.016), but it was also trending towards being less stiff than the ANT nail group (*p* = 0.052); however, there were no differences in stiffness between any nail groups (0.759 ≤ *p* ≤ 0.999).

For coronal stiffness, the INT group was less stiff than all nail groups (*p* < 0.001); however, there were no differences in stiffness between any nail groups (0.777 ≤ *p* ≤ 0.998).

For sagittal stiffness, the INT group was less stiff than all nail groups (*p* < 0.001); moreover, there were differences in stiffness for ANT vs. POS (*p* = 0.025) and POS-P (*p* = 0.047) nail groups, as well as between NEU vs. POS (*p* < 0.001) and POS-P (*p* < 0.001) nail groups.

For torsional stiffness, the INT group was less stiff than all nail groups (*p* ≤ 0.018); however, there were no differences in stiffness between any nail groups (0.103 ≤ *p* ≤ 0.999).

For proximal stress, the INT group had higher stress than all nail groups (*p* ≤ 0.027); however, there were no differences in stress between any nail groups (0.098 ≤ *p* ≤ 0.987). There was a trend of increasing proximal stress from ANT to NEU to POS to POS-P groups.

For distal stress, there were no differences between any group comparisons (*p* = 0.096). There was a trend of increasing distal stress from ANT to NEU to POS to POS-P groups.

## 4. Discussion

### 4.1. Comparison to Prior Work

Comparing current data to prior work can confirm the validity of results and place them in a broader context. Any divergences are due to different femur sizes and bone qualities, cephalomedullary nail designs, loading fixtures and protocols, rosette locations, etc. For instance, present INT osteoporotic femurs had an axial stiffness (300 ± 51 N/mm) (Figure 5(a)) similar to the initial design iteration of the same femurs (382 ± 61 N/mm) [31], as well as overlapping with intact cadaveric elderly femurs (365 ± 126 N/mm) [36] and osteoporotic femurs (419 ± 169 N/mm) [31]. Similarly, the current NEU group without a created fracture had an axial stiffness (434 ± 64 N/mm) (Figure 5(a)) that overlapped perfectly with prior studies using the same commercial nail that was also neutrally inserted despite being tested in larger artificial femurs with unstable peritrochanteric fractures (359 ± 107 N/mm to 525 ± 68 N/mm) [12, 13, 15], but was much less stiff than a similar long cephalomedullary nail tested in intact cadaveric femurs (729 ± 142 N/mm) [36]. Also, current axial tests produced proximal bone stresses for INT (3.8 ± 0.6 MPa) (Figure 5(e)) and NEU (2.4 ± 0.8 MPa) groups (Figure 5(e)) similar to a prior study [36] once its raw proximal strain gage data are load-matched to the current axial load (250 N) and converted to stress using human cortical bone elastic compressive modulus (17.4 GPa) [37], thereby yielding proximal bone stresses for intact cadaveric femurs (2.9 ± 0.8 MPa) and long cephalomedullary nails in the same intact femurs (3.2 ± 0.4 MPa).

### 4.2. Practical Implications

Present trends suggest only a marginal influence of femoral nail insertion point on construct stiffness, whereas the effect on bone stress could increase the risk of perforation and/or fracture, although most comparisons were not statistically significant. Specifically, maximum differences for ANT vs. NEU vs. POS groups for stiffness were only 9.9% (axial) (Figure 5(a)), 3.5% (coronal) (Figure 5(b)), 14.5% (sagittal) (Figure 5(c)), and 8.6% (torsional) (Figure 5(d)). However, for bone stress, maximum differences were 32.8% (proximal) (Figure 5(e)) and 30.7% (distal) (Figure 5(f)), such that stress rose as nail insertion point changed from ANT to NEU to POS causing the distal tip of the nail to gradually move closer to the anterior cortex to create a “stress riser,” thereby increasing the risk of perforation and/or fracture. Consider the case whereby these constructs are axially loaded with 10x more force to a clinical level of 2500 N (i.e., 3-4x body weight for a 70 kg person); assuming linearity, axial stiffnesses for the POS group would remain at 425 N/mm, but bone stresses would increase by 10x to 25 MPa (proximal) and 23 MPa (distal). Although these values are still far below this artificial cortical bone's ultimate failure stress (75.6 MPa) [30], there may have been bone locations not currently tested that would experience even higher stresses. Since the bone stress measurements were the lowest for anterior and neutral nail insertion point (Figures 5(e) and 5(f)), it is recommended that surgeons should use these nail insertion points to minimize the possibility of perforating the anterior cortex.

Current trends suggest that the presence of an accidentally induced anterior cortical perforation may only slightly increase the risk of distal femur fracture, especially since comparisons of bone stress did not reach statistical significance. In particular, going from the POS to the POS-P group showed consistent but only marginal decreases in overall construct stiffness by 3.6% (axial) (Figure 5(a)), 3.9% (coronal) (Figure 5(b)), 0.7% (sagittal) (Figure 5(c)), and 3.8% (torsional) (Figure 5(d)). According to the computed tomography rigidity analysis (CTRA) [35], a construct stiffness loss of 35% must occur for axial, bending, and torsional loading modes in the presence of a femoral defect before the risk of fracture rises statistically, suggesting that the present perforation would not be a clinical problem. Although going from the POS to the POS-P group showed a more notable increase in bone stress of 11.4% at the distal strain rosette above the perforation (Figure 5(f)), this “stress riser” effect may only be important during stair ascent, squatting, and sitting/rising from a chair during which the anterior femur has peak tensile loads; this is not a concern for stance weight bearing and normal gait patterns [38]. This is consistent with a case series examining distal locking of femoral nails that deliberately induced anterior cortical defects (15-20 mm width and 30-40 mm length) in the distal femur and only prescribed restricted weight bearing to patients, but found no fractures through the defects [39].

Although anterior cortical perforation of the distal femur during antegrade nailing only happens at a rate of <1% [20, 21], which supports the general findings of the current biomechanical analysis, there is still a finite risk of displaced supracondylar fracture requiring revision surgery [24]. Consequently, several authors describe ways of preventing it, such as bending the guidewire to allow the surgeon to direct it more posteriorly away from the anterior cortex [40, 41], using the starting guide pin or the distal locking drill bit as a blocking screw to direct the guidewire posterior in the femoral shaft [40, 42], or using as many as 5 bicortical Steinmann pins to guide the nail posteriorly [43]. Of course, there are other risk factors for accidental perforation beyond the control of the surgeon, like natural bowing of the femur which can have a radius of curvature from 52 to 203 cm [44, 45] and the built-in bowing of different cephalomedullary nail designs whose radius of curvature has decreased over the years to prevent perforation from 186 to 300 cm (in 2004) [46] to 127 to 200 cm (in 2016) [44]. Even with the decrease in radius of curvature of cephalomedullary implants, there will be a subset of patients who will still be at risk. If perforation occurs, it needs to be addressed either by restricting the patient to partial weight bearing [22, 23] or applying lateral locking plates [23].

### 4.3. Potential Limitations

Several drawbacks in the present study are typical of in vitro biomechanical studies, although their elimination would not likely change the relative performance of the test groups. Artificial femurs were used to represent osteoporotic bone; however, these analogs were previously validated against cadaveric osteoporotic femurs with good agreement for axial, 4-point bending, and torsional stiffness, as well as screw pullout force [31]. This study did not assess artificial femurs that represented normal bone quality, but only osteoporotic bone quality. Thus, this provided a “worst case scenario” for the biomechanical stability of the femur/nail construct. Future work should include testing femurs with normal bone quality since cortical perforations can also occur in such femurs. Fractures were not simulated in the femurs, thereby representing the postunion rather than the acute postsurgery situation; however, the rationale of this study was to provide “proof of principle” results that would not be restricted to a particular fracture pattern. Load to failure was not measured in any loading mode; the reason was that the authors had a limited supply of cephalomedullary nails that needed to be used multiple times without damaging them, and thus, only nondestructive loads could be used. Similarly, the current study used an axial load that was below 1 body weight, so the nail could be reused in multiple femurs. However, even if 1 body weight was used, this would have not changed the axial stiffness values measured because of the highly linear nature of the force-displacement graph. Axial stiffness would only be affected if extremely high loads were used just prior to mechanical failure but the relative axial stiffnesses between test groups would still be maintained for femur/nail construct [47]. Rosettes could only detect bone surface stresses at discrete locations; thus, future investigators may wish to employ imaging technologies (e.g., thermographic stress analysis or digital image correlation) that can provide full-field 3D stress maps to identify all potential “stress risers” [29].

## 5. Conclusion

This biomechanical study evaluated the influence in artificial osteoporotic femurs of changing the antegrade cephalomedullary nail insertion point from anterior to neutral to posterior locations relative to the tip of the greater trochanter with or without an anterior cortical perforation in the distal femur. This effect was only marginal on axial, coronal, sagittal, and torsional stiffness, but the increasing trends in bone stress may be of greater clinical importance because they are direct predictors of bone perforation and/or fracture. Considering the present bone stress measurements, it is recommended that surgeons should use anterior or neutral nail insertion points to reduce the risk of anterior cortical perforation.

## Data Availability

The data used to support the findings of this study are available from the corresponding author upon request.
